# Risk factors of acute renal failure in patients with protective ileostomy after rectal cancer surgery

**DOI:** 10.1186/s12893-023-02016-4

**Published:** 2023-04-28

**Authors:** Marghich Omar, Anis Tarek, Bouassria Abdeslam, Efoé-Ga Yawod Olivier Amouzou, Ousadden Abdelmalek, Ait Taleb Khalid, El Bouhaddouti Hicham, Mouaqit Ouadii, Mazaz Khalid, Rahili Mohamed Amine, Benjelloun El Bachir, Benizri Emmanuel

**Affiliations:** 1 Department of Visceral Surgery, University Hospital Hassan 2, Fes, Morocco; 2 General and Oncology Surgery Unit, Archet 2 Hospital, University Hospital of Nice, Nice, France; 3 Faculty of Medicine, Pharmacy and Dental Medicine, Sidi Mohamed Ben Abdellah University, Fes, Morocco

**Keywords:** Rectal cancer, Protective ileostomy, Risk factors, Acute renal failure

## Abstract

**Background:**

Despite the potential benefits of protective ileostomy in rectal surgery, diverting loop ileostomy construction is not free of specific medical consequences implying unplanned hospital readmissions. The most common reason for readmission in these patients is a dehydration with a prevalence of acute renal failure (ARF) of 20%. The objective of this study was to establish the predictive factors of ARF in patients with protective ileostomy after surgery for rectal cancer from a bicentric study.

**Methods:**

we conducted a bicentric retrospective cohort study to identify the risk factor of ARF. This study was carried out on 277 patients operated for rectal cancer with necessity of a protective ileostomy during the study period. ARF was measured at any endpoint between ileostomy creation and reversal. Multiple logistic regressions were performed to identify independent risk factors.

**Results:**

A total of 277 patients were included, and 18% (n = 50) were readmitted for ARF. In multivariate logistic regression, increased age (OR 1.02, p = 0.01), Psychiatric diseases (OR 4.33, p = 0.014), Angiotensin II receptor blockers (OR 5.15, p < 0.001) and the ASA score ≥ 3 (OR 9.5, p < 0.001) were significantly associated with ARF.

**Conclusion:**

Acute renal failure is a prevalent and significant event in the postoperative course of ileostomy patients. Patients at risk should be risk stratified before discharge and targeted for intensive preventive measures.

## Introduction

Colorectal cancer (CRC) is already the third leading cause of cancer death in the world, and its incidence is steadily rising in developing nations. The CRC is more incident among men than women and 3–4 times more common in developed than in developing nations. [1]

Surgery is the major treatment modality and is often supplemented by preoperative neoadjuvant radiotherapy and chemotherapy or postoperative adjuvant chemotherapy. [2]

Anterior resection of rectal cancer is the major anal-preservation operation. However, anastomotic leakage is one of the most important surgical complications of colorectal surgery, and it has been of great concern due to high occurrence of morbidity and mortality. The proximal diversion, either by a colostomy or an ileostomy, by preventing fecal flow through the anastomosis, minimizes the consequences of the anastomotic leak. [3]

The current trend favors protective ileostomies rather than colostomies in the protection of colorectal anastomose [4]; However, protective loop ileostomy was found to be associated with a high morbidity. In addition to local complications, the presence of an ileostomy “short-circuits” colic stool dehydration resulting in chronic water and sodium depletion. It is therefore not surprisingly, that the most common reason for readmission in these patients is a dehydration with a prevalence of acute renal failure (ARF) of 20% [5–10].

In order to establish strategies for the prevention of ARF within those patients it is necessary to determine the risk factors.

The objective of this study is to establish the predictive factors of ARF in patients with protective ileostomy after surgery for rectal cancer from a bicentric study.

## Methods

We carried out a bicentric retrospective cohort study, performed at the department of general surgery and digestive oncology of the university hospital of l’Archet 2 (Nice, France) and the department of general surgery of the university hospital Hassan II (Fez, Morocco).

Patients undergoing surgeries from January 2009 to December 2019 in the university hospital l’Archet 2 and from January 2015 to December 2020 in the university hospital Hassan II were retrospectively identified from the surgical act’s archives. Patients undergoing elective rectal resection with primary anastomosis and formation of a protective ileostomy were included in the study. Exclusion criteria were: pathology other than rectal adenocarcinoma, sigmoid colonic cancer and palliative loop ileostomy.

ARF, defined by the Acute Kidney Injury Network (AKIN) [8], was measured at any endpoint (outpatient consultation, readmission in surgery, emergency or acute care unit) between ileostomy creation and reversal.

Baseline data collected included: age, sex, cardiovascular risk factors and cardiovascular diseases, chronic alcoholism, chronic smoking, renal disease (defined as any nephropathy with or without decreased glomerular filtration rate), respiratory disease (asthma, chronic obstructive pulmonary disease, pulmonary tuberculosis), psychiatric diseases (dementia, depressive disorder), and long-term medication use. The ASA score, a comorbidity classification associated with post-operative morbidity and the Clavien-Dindo complication scoring system, classifying postoperative complications based on therapy required were calculated for each patient. Other variables such as neoadjuvant treatment, surgical approach and season of the procedure were collected.

### Statistical analysis

The data was stratified based on whether the patient presented ARF in the follow up.

The data were entered and coded on Excel, the statistical analysis was done in two steps.

− 1st step: We performed a descriptive analysis of the collected data. The results were presented in the form of percentages for the qualitative variables and means ± standard deviation for the quantitative variables.

− 2nd step: univariate analysis was used to determine the factors associated with acute renal failure using the Student and Chi 2 statistical tests then a multivariate analysis using stepwise multiple logistic regression was performed. The independent variables for the model were selected from univariate analysis based on a threshold p-value of 0.20. The results are reported in commented tables. A p < 0.05 was considered significant.

## Results

We report in this study 277 patients operated for rectal cancer with necessity of a protective ileostomy during the study period. 159 (57%) were male and the median age was 60.95 years ± 12.48 (range between 27 and 90 years). 50 of 277 patients (18%) were readmitted for ARF during their follow-up period prior to the stoma closure.

Diabetes and high blood pressure were the most common comorbidities within our patients, respectively 14.1% and 27.1%. Table 1.

**Table 1:** Comorbidities of the population in the study

Comorbidities	Number	Percentage
Diabetes	39	14.1
High blood pressure	75	27.1
Cardiovascular diseases	24	8.7
Respiratory diseases	18	6.5
Psychiatric diseases	18	6.5
Renal diseases	8	2.9
Chronic alcoholism	37	13.4
Chronic smoking	58	20.9
Dyslipidemia	34	12.3

Two hundred thirty-three of our patients (84.1%) had an ASA score < 3. Neo adjuvant treatment either by radio-chemotherapy or exclusive radiotherapy was initiated in 238(85.9%) of our patients. A laparoscopic approach was used in184(66.4%) of our patients and a laparotomy in 93(33.6%) of patients. 62 (22.5%) of our patients presented postoperative complications classified according to the Clavien-Dindo classification (Table 2).

**Table 2:** Post operative complications according to the Clavien-Dindo classification

Complications: Clavien-Dindo classification	Number	Percentage
All grades	62	22.5
Grade 1	27	9.7
Grade 2	12	4.4
Grade 3	23	8.4

Seventy-four of our patients (26.7%) were operated in autumn, 74 (26.7%) in winter, 90 (32.5%) in spring and 39 (14.1%) in summer.

Among the 55 patients readmitted for ARF, 15 patients (30%) progressed to chronic kidney disease and 4 patients (8%) have died.

Univariate analysis identified increased age, high blood pressure, diabetes, renal diseases, psychiatric diseases (dementia, depressive disorder), medications (diuretics, angiotensin II receptor blockers and ACE inhibitors) and ASA score ≥ 3 were significantly associated with an ARF in the follow up course. Table 3.

**Table 3:** Summary of the univariate analysis

	All patients N = 277	Acute renal failure N = 50(18.0%)	No acute renal failure N = 227(82.0%)	P			
**Sex**				P = 0.345			
M F	159(57.4%) 118(42.6%)	32(64.0%) 18(36.0%)	127(55.9%) 100(44.1%)				
**Median age**	60.95 ± 12.48	68.50 ± 12.17	59.29 ± 11.95	P = 0.001			
**Comorbidities**							
High blood pressure Diabetes Chronic smoking Dyslipidemia	75(27.1%) 39(14.1%) 58(20.9%) 34(12.3%)	27(54.0%) 14(28.0%) 12(24.0%) 9(18.0%)	48(21.1%) 25(11.0%) 46(20.3%) 25(11.0%)	P = 0.001 P = 0.003 P = 0.756 P = 0.231			
Chronic alcoholism	37(13.4%)	4(8.0%)	33(14.5%)	P = 0.259			
Psychiatric disease	18(6.5%)	8(16.0%)	10(4.4%)	P = 0.007			
Cardiovascular diseases	24(8.7%)	8(16.0%)	16(7.0%)	P = 0.082			
Respiratory disease	18(6.5%)	4(8.0%)	14(6.2%)	P = 0.750			
Renal disease	8(2.9%)	4(8.0%)	4(1.8%)	P = 0.038			
**Treatment**							
diuretics ACE inhibitors Calcium channel blockers angiotensin II receptor blockers	26(9.4%) 27(9.7%) 20(7.2%) 33(11.9%)	9(18.0%) 12(24.0%) 5(10.0%) 18(36.0%)	17(7.5%) 15(6.6%) 15(6.6%) 15(6.6%)	P = 0.037 P = 0.001 P = 0.545 P = 0.001			
**ASA score**				P = 0.001			
< 3 ≥ 3	233(84.1%) 44(15.9%)	24(48.0%) 26(52.0%)	209(92.1%) 18(7.9%)				
**Neo adjuvant treatment**	238(85.9%)	45(90%)	193(85%)	P = 0.386			
**Surgery**				P = 0.741			
Coelioscopic approach Laparotomy approach	184(66.4%) 93(33.6%)	32(64.0%) 18(36.0%)	152(67.0%) 75(33.0%)				
**Post operative complications**				P = 0.365			
Clavien-Dindo classification							
1 2 3	27(9.7%) 12(4.4%) 23(8.4%)	4(8.0%) 2(4.0%) 5(10.0%)	23(10.2%) 10(4.4%) 18(7.9%)				
**Season of the procedure**				P = 0.280			
Autumn Winter Spring Summer	74 (26,7%) 74 (26,7%) 90 (32,5%) 39 (14,1%)	12(24,0%) 10(20,0%) 22(44,0%) 6(12,0%)	62(27,3%) 64(28,2%) 68(30,0%) 33(14,5%)				

In multivariate analysis, increased age, the ASA score, psychiatric diseases and ARA 2 were independently associated with an acute renal failure. Table 4.

**Table 4:** Multiple Logistic Regression Results

	OR	95% CI	P
Age	1.02	0.98–1.05	P = 0.01
Psychiatric disease			P = 0.014
No yes	1 4.33	1 1.34-14.00	
Angiotensin II receptor blockers			P < 0.001
No Yes	1 5.15	1 2.08–12.74	
ASA score			P < 0.001
< 3 ≥ 3	1 9.50	1 4.32–20.87	

## Discussion

The objective of this study was to establish the predictive factors of acute renal failure in patients with protective ileostomy after surgery for rectal cancer in order to establish strategies for the prevention of patients at risk.

This study is, to our knowledge, one of the few cohorts studying the risk factors for the occurrence of renal failure after protective ileostomy for rectal cancer surgery.

The question has been treated in a different way by other researchers. Some have focused on readmission rates and the impact of ileostomy on kidney function [11–14].

The results of this study reinforce the findings of previous studies. Thus, our acute renal failure rate was 18%, a rate close to those of Gessler et al. [15] (21%) and Beck-Kaltenbach et al. [7] (30%).

The univariate analysis showed that increased age, high blood pressure, diabetes, renal diseases, psychiatric diseases (dementia, depressive disorder), medications (diuretics, angiotensin II receptor blockers and ACE inhibitors) and ASA score ≥ 3 were significantly associated with an acute renal failure. After the multivariate analysis, only increased age, ASA score, psychiatric diseases and angiotensin II receptor blockers were independently associated with an acute renal failure.

Age was the most identified risk factor responsible for acute renal failure in the literature as reported by Gessler [15] in his series published in 2014, paquette [11] in his series published in 2013 and Fiedeling [13] in his series published in 2020. For high blood pressure, only one study in the literature, that of Gessler [15], has shown its involvement in the occurrence of acute renal failure. In our study, high blood pressure did not reach the significance threshold. The use of antihypertensive drugs can be a source of readmission for ARF. This concerns ARA2, reported by Fiedeling [13] and diuretics reported by Messaris [5]. In our study, angiotensin II receptor blockers were significantly associated with readmissions for ARF. However, psychiatric diseases (dementia, depressive disorder) and an ASA score ≥ 3 represent to our knowledge new findings in the literature.

The season of the operative act, a variable studied for the first time in the literature to our knowledge, was studied to investigate whether the higher incidence of dehydration during summer months could exacerbate kidney damage but it was not associated with ARF in our research.

It should be highlighted that ARF is a serious condition that can have severe consequences for patients. Our study showed that among the 55 patients who were readmitted for ARF, 15 patients (30%) progressed to chronic kidney disease and 4 patients (8%) died. We did not find any data in the literature regarding the evolution of patients with ARF due to an ileostomy.

This study has multiple clinical implications, it will allow us to reinforce the attention needed for patients with ileostomies in their postoperative course both inside and outside the hospital and to establish prevention measures for high-risk patients and therefore it will allow us to improve the outcomes in this population.

Limitations of our study include the retrospective nature of the study and the fact that we did not measure ileostomy output or collect data about the use of loperamide; however, this study is one of the few cohorts studying the risk factors for the occurrence of renal failure after protective ileostomy for rectal cancer surgery.

### Conclusion

Acute renal failure is a prevalent and significant event in the postoperative course of ileostomy patients occurring in 18% of them. The causes are heterogeneous, but the most influential predictive factors appear to be the age, ASA score, psychiatric diseases, and use of ARA2. Patients at risk should be risk stratified before discharge and targeted for intensive preventive measures fighting dehydration; high stoma output and low water and electrolytes input.

## Data Availability

All data generated or analyzed during this study are included in this published article.

## List of Abbreviations

ACE: Angiotensin-Converting Enzyme
AKIN: Acute Kidney Injury Network
ARF: Acute renal failure
ASA: American Society of Anesthesiologists
CRC: Colorectal cancer
